# Exploring the relationship between vaccine hesitancy and mothers’ perspectives on COVID-19 vaccines for children ages 5–11 years during the omicron predominant period 2021–2022: a qualitative study

**DOI:** 10.3389/fpubh.2024.1355452

**Published:** 2024-07-08

**Authors:** Tiffany A. Suragh, David Adzrago, Marlyn A. Allicock, Paul G. Yeh, Paula Cuccaro

**Affiliations:** Department of Health Promotion and Behavioral Sciences, School of Public Health, The University of Texas Health Science Center at Houston (UTHealth), Houston, TX, United States

**Keywords:** COVID-19, childhood vaccination, vaccine hesitancy, vaccine refusal, qualitative

## Abstract

**Background:**

The United States Food and Drug Administration authorized COVID-19 vaccines for children ages 5–11 years in October 2021 during the Omicron predominant period. Parental vaccine hesitancy was prevalent during this time, resulting in low childhood COVID-19 vaccine uptake. Most studies exploring factors influencing parental vaccine hesitancy have focused on racial and ethnic minorities and lower socioeconomic populations; however, there is little knowledge of the drive drivers of vaccine hesitancy among White parents with higher education and socioeconomic statuses.

**Methods:**

We conducted semi-structured interviews with a sample of 15 White mothers of children ages 5–11 years in Atlanta, GA, between October–December 2021. Thematic analysis was performed using NVivo 12.

**Results:**

Mothers were college-educated, homeowners, and fully vaccinated against COVID-19. Key findings included decreased pediatrician’s recommendations for COVID-19 vaccines, reliance on information from specialized doctors and scientists, distrust in public health authorities, high risk-perception of COVID-19 vaccines, and low risk-perception of COVID-19 disease. Factors related to vaccine acceptance were altruism and practicality.

**Conclusion:**

This study adds to the sparse literature on reasons for vaccine hesitancy among White mothers of children ages 5–11 years with higher educational and socioeconomic status. Improving vaccine uptake among this group is critical for protecting the health of their children and other vulnerable populations. Tailored vaccine messaging and intervention are warranted to address their unique attitudes, beliefs, and behaviors. An enhanced understanding of the factors influencing subpopulations of parents can help vaccine policymakers and healthcare providers improve efforts to reduce vaccine hesitancy, particularly for new vaccines.

## 1 Introduction

The coronavirus disease (COVID-19) pandemic has highlighted the implications of vaccine hesitancy on low vaccine uptake and acceptance (1). It has demonstrated vaccine hesitancy’s threat to individual and population-level protection against highly infectious diseases (1, 2). Vaccine hesitancy – the delay or refusal of a vaccine despite its availability – is associated with low COVID-19 vaccine uptake among children (3–5). COVID-19 has resulted in negative health outcomes in children, including multisystem inflammatory syndrome (MIS-C). This has resulted in hospitalizations and deaths in children ages 5–11 years (6, 7). Nearly 15.6 million children in the United States (U.S.) have tested positive for COVID-19 (7), with the highest number of cases occurring during the Omicron predominant period 2021–2022. The FDA authorized the Pfizer-BioNTech COVID-19 vaccine for emergency use in children ages 5–11 years on October 29, 2021 (8). COVID-19 vaccination is the most effective way to reduce children’s COVID-19 morbidity and mortality, yet vaccination coverage remains suboptimal, with White children having some of the lowest vaccination rates (45%) compared to Asian and Hispanic Children (75 and 49%, respectively) (9). Geographic differences in vaccination coverage also exist, with eight of the 10 states having the lowest vaccination coverage in the South (10).

Vaccine hesitancy may explain the vaccination coverage disparities among children ages 5–11 years. Vaccine hesitancy strongly influences parents’ decisions to vaccinate their children and is a complex notion varying across vaccine types, times, and settings (3). The World Health Organization’s (WHO) Increasing Vaccination Model (IVM) proposes that vaccine uptake is based on people’s thoughts and feelings, such as perceived risks and benefits, and social processes, like social norms and information (11). In addition to these factors, practical issues such as vaccine cost and availability result in vaccine acceptance, delay, or refusal (11). Vaccine hesitancy is influenced by multiple interrelated factors, and some individuals may accept some vaccines and refuse or delay others (12).

Reasons for parental COVID-19 vaccine hesitancy included the vaccine’s novelty and lack of confidence in its safety and efficacy (13–18). For example, parents described that the vaccines were developed too quickly and were not well studied leading to concerns about unknown vaccine risks and potential side effects, such as myocarditis and fertility issues (14, 19, 20). The changing vaccine recommendations, including the need for boosters and vaccine dosage in children, also created confusion and doubt among parents regarding the necessity of childhood vaccinations (14–17, 21, 22). Studies found that vaccine-hesitant parents tended to have less knowledge of vaccines, leading to less confidence in the vaccine’s efficacy (19). Beliefs that COVID-19 was more severe in children with pre-existing conditions and adults led to vaccine hesitancy among parents who felt their children were healthy and did not feel compelled to protect the community at large (14–16). Distrust of government and public health officials also contributed to conspiracy theories and misinformation about the COVID-19 vaccine (13, 14, 17–20). This misinformation spread quickly through social media, where some parents seek vaccine information (18–20). These beliefs were persistent even among parents fully vaccinated against COVID-19 (13, 23).

Vaccine hesitancy is also largely influenced by demographics such as race, income, and educational level (24, 25). A common belief is that vaccine-hesitant individuals are of lower income and educational status and occur mostly among racial and ethnic minorities (13, 25, 26). However, vaccine hesitancy is prevalent among White individuals and those with higher educational levels and socioeconomic status (24, 27). This may be due to the environments in which they live and work, which might limit their exposure to large groups of people (e.g., crowded schools and houses) and influence their perceptions of disease severity. A Kaiser Family Foundation poll found that parents who are Black or Hispanic are less likely to feel their child is “very safe” from COVID-19 at school than White parents (33% vs. 52%) (28).

Many studies examining parental perceptions of childhood COVID-19 vaccines have been quantitative (13, 16, 21–23), with few qualitative studies (14, 15, 29). Qualitative research allows the gathering of rich data and exploration of thoughts and feelings that enable researchers to gain insight into decision-making processes and help explain human behavior, including a mother’s decision to vaccinate their child against COVID-19 (30). To our knowledge, this was one of the few qualitative studies to explore maternal perceptions of COVID-19 vaccines for children ages 5–11 immediately after the FDA introduced the vaccine. Lastly, most of the literature has focused on drivers of vaccine hesitancy among racial and ethnic minorities and those with lower socioeconomic status (31–33). Limited information exists on factors influencing COVID-19 hesitancy among White, higher-income, and educated parents. It is critical to increase vaccine uptake among this subpopulation to protect their children and other vulnerable groups and increase vaccine uptake.

The objective of the study was to understand the beliefs, attitudes, and behaviors of White mothers of high socioeconomic status and education levels regarding COVID-19 vaccination among children ages 5–11 years. Findings can have clinical and policy implications, such as better strategies to target different subpopulations of parents with unique vaccine hesitancy concerns and needs.

## 2 Methods

### 2.1 Participants

We conducted semi-structured interviews with 15 mothers from October to December 2021. We used purposive sampling to recruit mothers. The sample was recruited primarily from one childcare service/company that connects families with in-home care in Atlanta, GA, and the rest of the sample was obtained through snowball sampling. The lead author emailed the childcare service describing the study and asked if the information could be passed to mothers. After each interview was completed, the lead author asked the participant to share information about the study with other mothers who might be interested. Mothers were eligible to participate if they had a child ages 5–11 years, lived in Atlanta, GA, and spoke English.

### 2.2 Data collection

We used semi-structured interviews to create dialog while allowing for emergent ideas (34, 35). We developed a semi-structured interview guide based on a literature review and consultation with experts in qualitative research and vaccine hesitancy, including faculty at the University of Texas Health Science Center at Houston (UTHealth Houston). Interview questions were not tied to a particular theory or framework to allow researchers to gather rich, complex data without being constrained by theoretical constructs. Mothers were asked broad, open-ended questions about their perceptions of routine and COVID-19 childhood vaccines. Questions centered around their views of COVID-19 vaccines and how this compared to routine childhood vaccines, trusted sources of COVID-19 vaccine information, perceptions of COVID-19 disease, and factors that would influence their decision to vaccinate their child against COVID-19. Verbal consent was received before the interviews. Interviews lasted 45–60 min and were audio and video recorded using Zoom (Zoom Video Communications Inc., 2016). Interviews were professionally transcribed verbatim, and transcripts were independently coded by a primary (TS) and secondary (DA) reviewer. A third reviewer resolved disagreements of codes (PC).

### 2.3 Data analysis

Thematic analysis was performed and included deductive and inductive approaches using NVivo 12 software (released in March 2020) (36). Utilizing Braun and Clarke’s thematic content analytic approach, the researchers (TS & PC) first conducted multiple transcript readings that were discussed among the research team to develop a codebook that aligned with the research question and study objective (37, 38). Emergent themes were discussed among the researchers and used in revising the codebook. Analysis continued with the refinement of codes until saturation was met and no new codes or themes emerged (39). All study procedures were approved by the Committee for the Protection of Human Subjects (the institutional review board) at UTHealth Houston (HSC-SPH-11-0577).

## 3 Results

### 3.1 Sample

In total, 15 mothers with children ages 5–11 years completed individual interviews. Participants were White, college-educated homeowners and the majority reported receiving two doses of COVID-19 vaccines for themselves. Participants’ ages ranged from 36 to 47 years (Table 1). Pseudonyms were used to describe the participants to protect the identity of the participants and because the sample was demographically similar (40). The majority of mothers were hesitant about COVID-19 vaccines, and their hesitancy was driven by the unfamiliarity and novelty of the vaccine, balancing the risks and benefits of the vaccine, distrust in government, science, and public health authorities, decreased trust in pediatricians and increased trust in medical specialists. Mothers who were accepting of COVID-19 vaccines had a greater fear of adverse effects of COVID-19 disease than the vaccine and wanted to return to normalcy with children in school and protect the greater community. The following themes emerged in relation to COVID-19 vaccine hesitancy and vaccine acceptance:

**Table 1:** Demographic characteristics of mothers of children ages 5–11 years (*N* = 15).^*^

**Demographics**	***N* (%)**	**%**
**Age (years)**		
29–39	10	67
40–49	5	33
**Education**		
Bachelor’s degree	5	33
Graduate degree	10	67
**Number of children**		
1–2	11	73
3–4	4	27

*Mothers were all White, owned their homes and were vaccinated against COVID-19.

### 3.2 Vaccine hesitancy is influenced by the unfamiliarity and novelty of the vaccine

Mothers described their unwillingness to vaccinate their children based on their level of familiarity and novelty surrounding COVID-19 vaccines. Routine vaccines for known illnesses such as influenza were viewed differently from emergent diseases like COVID-19. For routine childhood vaccines like the Rotavirus vaccine, mothers described confidence in the vaccines’ safety and efficacy due to the length of time the vaccines had been around and having been vaccinated themselves as a child. This familiarity resulted in mothers’ confidence and acceptance of routine vaccines as recommended by their pediatricians. Conversely, uncertainty and fear led to the nonacceptance of childhood COVID-19 vaccines. Mothers expressed the vaccine’s novelty, fast development, and unknown long-term side effects as reasons for low confidence in the vaccine. Paradoxically, mothers who were vaccinated against COVID-19 unwilling to vaccinate their children, and hesitant to follow their pediatrician’s recommendations as they normally would before the COVID-19 vaccine. Lauren expressed her views on routine and COVID-19 vaccines,

… I pretty much just followed the standard doctor’s guidelines. So, whenever…we had a doctor’s appointment when they were little kids…. I always consented to them to get the vaccines. I never delayed…the vaccines that we have done in the past have been around for such a long time, and so there’s a lot of, you know, evidence that they are not harmful. And with…these [COVID-19] vaccines, they have come out so quickly, gotten through the approval process so quickly (Lauren, mother of 2 children).

The fast approval and quick manufacturing of COVID-19 vaccines made mothers wonder if the vaccine risks outweighed the benefits.

### 3.3 Balancing the risks and benefits of childhood COVID-19 vaccination

Some reasons underlying mothers’ hesitancy towards COVID-19 vaccines were due to unknown and potential long-term side effects, particularly their novelty and the short manufacturing times for these vaccines. Maria expressed concerns about possible side effects,

I think it’s probably effective and safe. Because I feel like people, millions of people, hopefully, would not be taking it if it wasn’t. I guess because it’s so new, we do not know what any of the long-term side effects are. Like, is my son going to have fertility issues later? Is it going to affect his sperm? (Maria, mother of 2 children).

These concerns outweighed any perceived benefit to vaccination. The belief that vaccinating children would protect them and the community did not motivate mothers toward vaccination. Rachel expressed her ambivalence about COVID-19 vaccines,

I think it is one of the hardest decisions that I’ve ever had to make for my kids… we are not set on which way we’ll go right now…you just do not want to do anything to harm your kids…. And this, I’m not convinced we are doing it for the kids yet and that makes me really nervous, and I do not want to make a bad decision for them…because it could affect them for the rest of their life. And there’s just so much unknown (Rachel, mother of 2 children).

A sentiment expressed by mothers was that their children were not at risk for severe complications from COVID-19 because they were healthy and unexposed to large groups. Some mothers described having pods of 4–5 children that studied and played together when schools were closed and not having to be around many people. Olivia shared her reasons for waiting to vaccinate,

My children, luckily, do not have any problems other than being preemies. They are not obese. There are no tobacco users in our life. We’re very thankful not to have any of those sorts of risk factors. We also live in Atlanta, where we do not ride public transportation on a regular basis…we are not exposed to big crowds, that kind of thing. It might be different if I was taking a subway in New York City every day and come in contact with a lot of people, but, my plan is to wait at least two years… (Olivia, mother of 2 children).

### 3.4 Vaccine hesitancy influenced by distrust science

Vaccine hesitancy was influenced by distrust in government and public health officials and increased trust in medical and infectious disease specialists.

Mothers who were hesitant about childhood COVID-19 vaccines described their lack of confidence and trust in the scientific information disseminated to the public by the government, vaccine manufacturers, and public health officials. They were skeptical about the vaccine’s safety and efficacy due to the perceived lack of rigor in the scientific studies. Mothers did not think there was enough evidence to justify childhood COVID-19 vaccination and were not convinced vaccination was the decision for their children. Their ability to interpret scientific information independently influenced their trust and confidence in science.

Sarah expressed her doubts,

…this experience has dramatically changed my perspective on our medical institutions and the amount of trust I have in them in a major way…The sample size with kids for the Pfizer testing was…roughly, like, a little under 2,200. That, to me, is not a large enough sample with a large enough time period for me to sign my kid up… (Sarah, mother of 3 children).

The changing and conflicting messaging by the public health authorities during the COVID-19 pandemic contributed to mothers’ uncertainty about the vaccine. Messages, like the length of time children needed to wait to be vaccinated after having COVID-19, the spacing of COVID-19 and other childhood vaccines, and face mask guidelines led to mothers’ doubting the accuracy of information channeled through traditional sources, including their pediatricians. Despite having established relationships with their pediatricians, mothers now felt them to be less trustworthy and knowledgeable of the efficacy and safety of childhood COVID-19 vaccines. Ashley stated,

“…our doctor has been sending out emails every week about how safe the vaccine is. And I mean that’s great, but. And like how safe masking is for kids, but I just do not really, um, it’s just not to my taste. So, probably not her doctor…there are some news outlets that I find to be credible…” (Ashley, mother of 4 children).

Alternatively, mothers relied on information from specialty doctors like cardiologists and infectious disease specialists. Unlike pediatricians, whom they felt would recommend the vaccine regardless of potential safety concerns, mothers felt specialists provided more honest and independent reviews of childhood COVID-19 vaccines. For example, mothers felt more comfortable speaking to cardiologists regarding the potential risk of heart inflammation (e.g., myocarditis and pericarditis) following COVID-19 vaccination. This reliance on specialists was facilitated by the proximity and access to these professionals, who were their neighbors, friends, and family members. Maria expressed her reasons for increased reliance on specialist doctors, “…I love their pediatrician, and we have a really good relationship with him…I feel like the epidemiologist is closer to this specific focus…I know my doctor is not in the lab testing vaccines…he’s not on the front lines.” (Maria, mother of 2 children). This shifting trust in science influenced mothers’ perception of the severity of COVID-19 in children and the risks associated with the COVID-19 vaccine. Mothers did not mention reliance on specialists for routine vaccines, and it appears this shift in perception was related to the novelty and unfamiliarity of the COVID-19 vaccine as well as based on their trusted sources of vaccine information.

### 3.5 Vaccine hesitancy influenced by trust in source of COVID-19 vaccination information

A key driver of vaccine hesitancy among mothers was their level of trust in the sources of information promoting childhood COVID-19 vaccination. If mothers did not trust the information or agenda of institutions and authorities, they were less confident that the vaccine was the best decision for their children. Sources deemed trustworthy included scientific websites and organizations (e.g., the Mayo Clinic), news, podcasts, cardiologists, epidemiologists, family, and friends. While mothers mentioned viewing information on social media platforms like Facebook, they did not consider these platforms trustworthy or their primary source for vaccine information. They described the extent to which they would actively seek out information and make their conclusions about the vaccine (e.g., searching for adverse events following COVID-19 vaccination on publicly available government databases) instead of passively accepting information. Sarah described how she searched for credible information,

… I read the New York Times every morning, and I read the AJC [The Atlanta Journal-Constitution] every morning…I also, you know, Google things. And when I Google things, I try to click websites that I recognize and that are things like John Hopkins, Mayo Clinic…things that seem like they would not get away with misinformation… (Sarah, mother of 2 children).

Mothers were also keenly aware that politics heavily influenced the dissemination of COVID-19 vaccine information. They described the differing opinions regarding childhood COVID-19 vaccines’ safety and necessity based on conservative versus liberal sources of information. The politicization of the COVID-19 pandemic led mothers to question the true motivations behind campaigns promoting childhood COVID-19 vaccinations and whether vaccines were being used for political gain rather than to protect their children’s health. This perception contributed to mothers’ reluctance to vaccinate as they refused to put their children at risk for unknown side effects due to political pressure, creating doubt about the veracity of the scientific information being used to promote the vaccine for children. Rachel expressed her concerns,

…I do not trust the politics, and some of [the] encouragement to give it to them [children] is to protect adults who refused to get it themselves. And that’s not fair. I do not want to do something to my kids that the studies have not been fully done on to protect adults, who are old enough to decide for themselves and have the free choice to do it or not, and it’s their fault if they do not. I cannot help that in my opinion. And I’m not gonna punish my kids because they [adults] do not [get vaccinated] (Rachel, mother of 2 children).

### 3.6 Vaccine acceptance influenced by fear of COVID-19 disease and wanting to return to normalcy

The belief that COVID-19 vaccines could protect children and the community resonated with some mothers and affected their decision to vaccinate their children. Mothers who already vaccinated their children against COVID-19, or intended to, believed the benefits of vaccination outweighed the potential long-term risks of COVID-19 disease. Katherine expressed her concerns,

…I’m really nervous about the long-term effects of my son getting COVID because…Like, what if it causes some lung issues and he’s not able to play soccer like he does…we kind of know right now from the trials that there’s not going to be these really long long-term effects from the COVID vaccine, whereas I do not feel like we know that for actual COVID-19 infection…I do not think the COVID vaccine is going to make my son suffer, but I do feel like COVID, potentially, in the long run, could make him suffer…” (Katherine, mother of 1 child).

A minority of mothers accepted the vaccine for reasons like fewer missed school days and the ability to travel. They described older family members as more at risk for severe complications of COVID-19, and vaccinating their children was a way to allow them to spend time with others safely. For these women, altruism and practical reasons outweighed the perceived risks associated with the vaccine.

Allison expressed her considerations,

… besides the health, there’s also the logistics. Like I feel like I work in a hospital, and they are at risk, and so I wanted them protected. There’s also, at the schools, I know that there will be different requirements for quarantining if there’s exposures, and if they have vaccines, then they will not have to quarantine, so there will not be missed school…and all of that was really important to me. I do not want my kids learning virtually anymore, ever, if possible… (Allison, mother of 2 children).

Regarding the logistics of traveling, Lauren stated,

…we have some international travel coming up, so…we said, “Okay. Well, maybe we should go ahead and get the kids vaccinated…And it’s honestly less for the fact that you know, we think it’ll prevent them from contracting [COVID-19 infection] or their symptoms, but more from the fact of, you know, we do not want any disruptions to the travel, to be quite honest… (Lauren, mother of 2 children).

## 4 Discussion

Our study identified key factors related to vaccine hesitancy and acceptance of childhood COVID-19 vaccines among White, mothers of children ages 5–11 years from higher educational and socioeconomic backgrounds. Mothers who were vaccine-hesitant expressed unfamiliarity and fear of unknown adverse effects of COVID-19 vaccines, decreased trust in pediatricians’ recommendations, and distrust in science and public health authorities. Mothers who were vaccine-acceptant expressed sentiments including trust in the science and public health authorities, fear of COVID-19, and aspects of altruism such as wanting to protect their children, family, and community. Reasons for practicality and wanting to return to normalcy were also noted for accepting childhood COVID-19 vaccines.

Our findings reinforce the notion that vaccine hesitancy is a complex phenomenon and vaccine decision-making is influenced by many contextual factors (3, 11, 12). Individuals cannot be grouped exclusively as “hesitant” or “acceptant.” Mothers in our study were hesitant to vaccinate their children with COVID-19 vaccines yet accepted routine childhood vaccines like polio and influenza. Mothers’ familiarity with routine vaccines, such as having taken these vaccines as children and understanding their safety profiles, influenced their decisions to vaccinate their children. This finding aligns with research that found parents accepting some but not all vaccines and mothers vaccinated against COVID-19, yet still hesitant for their children (12, 13, 23). The novelty of COVID-19 vaccines routine vaccines made mothers feel less confident in the vaccines’ safety and efficacy (13–18). Similar to other studies, most of the mothers in our study were fully vaccinated against COVID-19 but were still concerned about the vaccine’s safety in their children, for example, citing long-term fertility issues (13, 14).

Our findings align with other vaccine trends that have shown demographic factors, including White, affluent, educated parents who are least likely to vaccinate their child against the human papillomavirus (HPV) and are a major group within the anti-vaccine movement (41–43). Despite this subpopulation having fewer potential barriers to vaccination (e.g., financial instability and lower education), their decision-making is influenced by their research, weighing the benefits and risks of vaccination, and personal beliefs values, and attitudes (44–46). In our study, mothers accessed, reviewed, and interpreted publicly available government datasets on adverse events following childhood COVID-19 vaccination to help them understand the vaccine’s safety and side effects. For example, mothers who believed the sample sizes of the clinical trials were small may feel more confident in their decision to vaccinate their child if they received detailed information on how to interpret sample size calculations and vaccine efficacy (45). This misunderstanding of COVID-19 scientific information being disseminated by the government and public health officials led to distrust in these entities and is a common theme in the literature. It is essential for policymakers to increase efforts to develop tailored materials that offer more transparent vaccine safety information for a subgroup of parents who are well-informed and access resources that most parents might not know exist (e.g., publicly available government surveillance databases). For example, vaccine messaging can include more technical information, such as how to interpret scientific data from clinical trials and government vaccine surveillance systems (44–46). These efforts can increase vaccine confidence and trust among parents.

Studies have found pediatric healthcare providers to be the most trusted sources of vaccine information and facilitators of childhood vaccine uptake (14, 21, 22, 44). We found contrasting evidence as mothers in our study expressed decreased trust in their pediatricians and increased reliance on medical specialists like cardiologists and epidemiologists for COVID-19 vaccine information. During the H1N1 pandemic, individuals also lost trust in the government and health authorities, decreasing their willingness to get vaccinated (47). Parental vaccine hesitancy is influenced by many factors, such as vaccine type and how it was introduced (e.g., during a pandemic or mass vaccination campaign) (44–46). It is essential to strengthen long-standing relationships between pediatricians and parents, especially during a pandemic, when parents may be more concerned and less trusting of scientific information. Policymakers can consider how to develop tools to encourage collaborative provider-parent communication. Creating an environment where parents feel confident in the information received as opposed to feeling pressured to vaccinate their child against their will or despite their concerns can be beneficial among subgroups of parents like those in our study (45, 48).

The politicization of COVID-19 vaccines influenced mothers’ hesitancy towards vaccinating their children against COVID-19. Mothers expressed distrust in the true motivations of public health authorities recommending childhood COVID-19 vaccination. The belief that politicians misused COVID-19 vaccine information for their political gains aligned with research that suggested public confidence in COVID-19 vaccines was affected by how the government handled the COVID-19 pandemic (49). A poll by the Kaiser Family Foundation found political affiliation to be a stronger predictor of whether someone is vaccinated than demographic factors, such as education, race, and age (50). Considering how social, contextual and political factors may influence vaccine attitudes and beliefs, highlights the importance of public health officials leveraging multiple communication channels to address parental concerns (51).

Mothers in our study mentioned podcasts and medical websites as trusted sources of information. Podcasts were preferred because they offered differing views of childhood vaccination, and mothers appreciated the neutrality of discussions. This neutrality countered the partisan bias typically found in traditionally conservative and liberal news. Interestingly, social media was not regarded as a source of vaccine information but rather just a place to socialize, contrasting the mounting evidence that misinformation on social media influences vaccine decision-making (18, 20, 52). For mothers similar to our sample, it may be more effective to disseminate public health messaging regarding childhood COVID-19 vaccination through outlets not related to social media.

Our findings also highlighted the nuances in parental and child COVID-19 vaccination status. While other studies found parents vaccinated or intended to vaccinate against COVID-19 also intended to vaccinate their children (13, 16), our study yielded different findings. The majority of mothers in our study were all vaccinated against COVID-19 and accepted the risks of vaccination for themselves. Yet some were still hesitant and unwilling to put their children at similar risk. Children ages 5–11 years are different than older age groups, such as adolescents who may have some autonomy around vaccine decision-making (15). Therefore, mothers of young children may feel an even greater responsibility for their child’s health.

Similar to other studies, parental motivations for vaccinating their child against COVID-19 included a desire to protect the broader community, return to normalcy, and mitigate the negative social and emotional consequences of COVID-19, such as educational losses due to missed school (14, 16). Mothers expressed getting their children vaccinated so they could travel internationally, and it would appear that the familial and logistical benefits of vaccination would outweigh any perceived risks associated with the vaccine. Mothers in our sample were highly affluent and may have different motivations for wanting their child vaccinated than less affluent mothers Also, policymakers may want to emphasize the importance of school-based interventions and immunization policies that can encourage vaccine uptake in children (53).

Vaccine hesitancy can be conceptualized as linear stages of hesitancy and non-hesitancy based on the Increasing Vaccination Model and the Precaution Adoption Process Model (11, 54, 55). Some mothers in our study would fall within the stages of undecided (i.e., considered but not yet decided) and refuse (i.e., considered and decided to refuse). A notable difference with our findings is that the process may not be linear. For example, some mothers immediately refused childhood COVID-19 vaccines without first being undecided. This behavior highlights the complexities of understanding vaccine hesitancy. There is a public health need to shift mothers from undecided to decided, and understanding the perspectives of mothers who may have fewer financial barriers and access to more resources, such as those who are highly educated, can help in tailoring policy-driven interventions. For mothers who accepted COVID-19 vaccines, according to the model above, some would still be classified as “decided but not yet highly educated” due to delays in getting their child vaccinated. These mothers described technical barriers like being put on a waitlist to see their doctor. Despite the variety of places where COVID-19 vaccination is available, it is possible that mothers feel more comfortable vaccinating their children in a pediatrician’s office or a school setting. This preference may inform policy decisions related to vaccine distribution and understanding how these barriers may be reduced to facilitate faster vaccination for willing parents.

### 4.1 Strengths and limitations

Our qualitative approach allowed for a deeper exploration of White mothers of higher educational and socioeconomic statuses’ perspectives, producing rich data that offer more context than quantitative survey data. While studies have highlighted influencers of vaccine hesitancy among minority populations, very few have focused on higher socioeconomic populations. We found mothers with higher educational and financial status hesitant to vaccinate their children ages 5–11 years against COVID-19. This finding suggests that parents of various backgrounds have different beliefs and values, and future public health efforts should consider this when promoting vaccine uptake. These differences are especially critical during the early months of a vaccine’s rollout during a pandemic when many lives are at risk (47, 56, 57). Our qualitative study is one of the few studies that occurred when the FDA authorized the Pfizer-BioNTech COVID-19 vaccine for emergency use in children ages 5–11 years and represented early views and perceptions. Additionally, COVID-19 vaccine uptake in children ages 5–11 years remains suboptimal, with White children having the third lowest vaccination coverage. These findings may help explain why White parents of higher educational and socioeconomic status might still be hesitant.

Limitations of our study include that our sample was purposively recruited from one setting, a childcare service, and represents one geographic location, Atlanta, GA. The sample comprised of White, highly educated, higher-income mothers from the South. Their perspectives are not generalizable to other mothers. We focused on mothers because they tend to be primary healthcare decision-makers for children, including vaccinations (41); however, not having the perspectives of fathers or other caregivers limits the conclusions we can draw from our findings. Our study took place at the beginning of the vaccine rollout for children ages 5–11 years, and it is possible that mothers’ perceptions changed over time.

### 4.2 Future recommendations

Our study demonstrates the need to develop tailored public health interventions, vaccine policies, and clear and transparent communication strategies to address the unique needs and concerns of subgroups of parents. Understanding how demographic factors, such as race, income, and education, may influence vaccine hesitancy may inform different strategies to shift attitudes and beliefs toward accepting childhood COVID-19 vaccines. Mothers in our study were informed about childhood COVID-19 vaccines by researching scientific websites, reading clinical trial information, talking to medical specialists who were their friends and families, and reviewing data from government surveillance databases. Developing an intervention that could connect mothers with medical and infectious disease specialists and scientists may be beneficial in addressing their concerns and countering misinformation. These interventions could potentially assist mothers with interpreting scientific information accurately, including understanding the limitations of surveillance systems. Mothers in our study did not rely on social media as trusted sources of information, and therefore, we recommend promoting vaccine information and engaging with parents through their preferred communication channels, such as podcasts. Future research is warranted to study vaccine hesitancy perspectives of other parents with higher education and socioeconomic statuses and from different geographical regions to see if similar patterns exist.

## 5 Conclusion

Vaccine hesitancy may influence a mother’s decision to delay or refuse to vaccinate their child against COVID-19 and may be influenced by demographic factors such as race, income, and educational attainment. Our study highlighted the unique vaccine concerns and needs of White mothers with higher educational and, socioeconomic status an understudied group. Most studies have focused on the driver of parental vaccine hesitancy among racial and ethnic minorities and those with lower socioeconomic status. Mothers in our study conducted their own research and reached out to members of their social network who were medical specialists such as cardiologists. They preferred discussing vaccine safety information, such as the potential long-term effects, with these specialists rather than their pediatricians and primary care providers. This is concerning, given that previous studies have found that recommendations from pediatricians are the most impactful on vaccine receipt (54). It suggests the need for more tailored vaccine communication strategies and interventions, such as connecting these parents with scientists, epidemiologists, and medical specialists who can answer their questions and help them understand the risk–benefit ratio of COVID-19 vaccines. Our sample did not rely on social media for vaccine information, and policymakers may need to increase their presence on alternative platforms, such as podcasts, to counter misinformation. Vaccine-hesitant parents are a heterogeneous group, and understanding the beliefs, attitudes, and behaviors of subpopulations of parents is critical in reducing vaccine hesitancy and increasing COVID-19 vaccine uptake. Findings from our study revealed initial perceptions of childhood COVID-19 vaccines for children ages 5–11 years and can inform vaccination policies and health promotion guidelines surrounding the introduction of novel vaccines for emerging diseases.

## Data availability statement

The datasets presented in this article are not readily available because it consists of audio and video recordings of interviews with participants. We want to protect the confidentiality and privacy of the participants. Upon request we may be able to provide summaries. Requests to access the datasets should be directed to TS, tiffany.suragh@uth.tmc.edu.

## Ethics statement

The studies involving humans were approved by the Institutional review board at UTHealth Houston (HSC-SPH-11-0577). The studies were conducted in accordance with the local legislation and institutional requirements. The participants provided their written informed consent to participate in this study.

## Author contributions

TS: Conceptualization, Data curation, Formal analysis, Investigation, Methodology, Writing – original draft, Writing – review & editing. DA: Formal analysis, Writing – review & editing. PY: Writing – review & editing. MA: Conceptualization, Formal analysis, Supervision, Writing – review & editing. PC: Conceptualization, Formal analysis, Methodology, Resources, Supervision, Writing – review & editing.
